# *Yuk-Mi-Jihwang-Tang*, a Traditional Korean Multiple Herbal Formulae, Improves Hippocampal Memory on Scopolamine Injection-Induced Amnesia Model of C57BL/6 Mice

**DOI:** 10.1155/2018/2821040

**Published:** 2018-01-04

**Authors:** Hye-Lim Lee, Sung-Ah Lim, Hye-Won Lee, Ho-Ryong Yoo, Hyeong-Geug Kim

**Affiliations:** ^1^College of Korean Medicine, Gachon University, Gyeonggi, Seongnam 13120, Republic of Korea; ^2^Department of Beauty and Health-Care, College of Public Health Care, Daejeon University, 62 Daehak-ro, Dong-gu, Daejeon 300-716, Republic of Korea; ^3^TKM-Based Herbal Drug Research Group, Korea Institute of Oriental Medicine, Daejeon 305-811, Republic of Korea; ^4^Department of Neurologic Disorders & Aging Brain Constitution, Dunsan Hospital, Daejeon University, 1136 Dunsan-dong, Seo-gu, Daejeon 302-122, Republic of Korea; ^5^Department of Biochemistry and Molecular Biology, Indiana University School of Medicine, Indianapolis, IN, USA

## Abstract

We evaluated neuropharmacological properties of Yuk-Mi-Jihwang-Tang (YJT) against scopolamine injection-induced memory impairment mice model. Mice were orally administered with YJT (50, 100, or 200 mg/kg) or tacrine (TAC, 12.5 mg/kg) for 10 days. At the first day of Morris water maze task, scopolamine (2 mg/kg) was intraperitoneally injected before 30 min of it. The hippocampal memory function was determined by the Morris water maze task for 5 days consecutively. Scopolamine drastically increased escape latency and decreased time spent in target quadrant. Pretreatment YJT properly improved them. Regarding the redox status, YJT significantly reduced the oxidative stress and it also exerted much effort to improve both superoxide dismutase and catalase activities in hippocampal gene expression and protein levels. These effects were well coincided with immunohistochemical analysis of 4-hydroxyneal-positive signals in hippocampal areas. Additionally, acetylcholine esterase activities and brain-derived neurotrophic factor abnormalities in the hippocampal protein levels were significantly normalized by YJT, and their related molecules were also improved. The neuronal proliferation in hippocampal regions was markedly inhibited by scopolamine, whereas YJT notably recovered them. Collectively, YJT exerts much effort to enhance memorial functions through improving redox status homeostasis and partially regulates acetylcholine esterase activities as well as neuronal cell proliferation.

## 1. Introduction

Based on Traditional Korean Medicine (TKM) clinical practices over a thousand years, there are strong evidences of effects that various herbal medicines have on intractable diseases, especially neurodegenerative diseases [1–3]. Regarding the neurodegenerative diseases, Alzheimer's disease and Parkinson's disease have become a critical medical issue in the world recently, particularly aging population [4–6]. According to the previous study, approximately around 34 million people have suffered from Alzheimer's disease, and among them 5 ≥ million people from the United States have been diagnosed as Alzheimer's disease patients [7].

Additionally, the pathophysiological features of neurodegenerative diseases are clinically progressed and developed by loss of cognitive abilities, which affects learning and memory dysfunction [8, 9]. Deposition of amyloid plaques, tau protein aggregation, cerebral oxidative stress, neuroinflammation, and cholinergic dysfunction were mainly accompanied with neurodegenerative diseases, and they can lead to psychological and pathophysiological complications such as anxiety, depression, concentration problems, and motor disturbances [10, 11]. Among the various symptoms of them, the memory impairment is mainly provoked by cholinergic system abnormality that involves cholinergic neurons, neurotransmitters, and their receptors [12]. The etiology and pathogenesis of neurodegenerative disorder, however, remain unclear till recent days. Among the various regions of brain tissue, only some parts revealed that the cholinergic dysfunctions are attributed to the loss of cholinergic neurons in the basal fore brain and hippocampus impairs cognitive ability [13, 14].

In the normal status, the cholinergic activity in the central nervous system (CNS) leads to contribution of hippocampal neuronal genesis and memory improvement through the cAMP response element-binding protein/brain-derived neurotrophic factor (CREB/BDNF) signaling pathways [15]. According to above reasons, the primary accessible treatment in clinic used the modulations of acetylcholinesterase (AChE) inhibitors such as tacrine or donepezil, which increase the availability of acetylcholine at cholinergic synapses [16]. This drug, which is thought to be a potent treatment on the neurodegeneration related therapeutics, however, is still needed to prove its efficacy clinically.

Contrary to western medicine, the TKM has recognized that neurodegenerative diseases were frequently aroused due to an imbalance of qi and blood flows, which are main factors of the human body. Among the various herbal medicines,* Yuk-Mi-Jihwang-Tang* (YJT), which is well known to a representative Korean Traditional herbal medicine, has been popularly used for patients with various disorders including aging-related disorders, obesity, ischemia, and immune suppression for hundreds of years in South Korea [17–19]. Particularly, the YJT showed its pharmacological properties on the aging-related diseases, especially enhancement of memorial function evidenced by scientific experiments until recent days [20]. However, there is no study about its therapeutic efficacies against AChE inhibitors of memory deficits model.

Thus, in the present study we investigated the antiamnesic effects of YJT on memory deficits in a mouse model of cognitive impairment by Ach system abnormality which is induced by single injection of scopolamine using mice model.

## 2. Materials and Methods

### 2.1. Preparation of YJT

YJT comprises 6 kinds of herbs including* Prepared Rehmannia glutinosa* Liboschitz* var. purpurea* Makino,* Dioscorea japonica* Thunb.,* Cornus officinalis* Sieb. et Zucc.,* Paeonia moutan* Sims,* Alpinia oxyphylla* Miq., and* Schisandra chinensis* Baill.; all these herbs were mixed with differential ratio of 4 : 2 : 2 : 1.5 : 1.5 : 1 (Table 1). All the herbal plants were obtained from the Dunsan Oriental Hospital of Daejeon University with inspection of Herbology professor (Daejeon, Republic of Korea). The herbal mixtures (total weights were 600 g) were boiled with distilled water (DW) at 100°C for 4 h and then filtered using a 300 mesh filter (50 *μ*m). After condensing the extract for 1 h, it was placed under −70°C for at least 3 h. The frozen extract was processed to the frozen lyophilization for 72 h and collected them and weighed. The final yield was 15.51% (w/w).

### 2.2. Fingerprinting Analysis of YJT

**Figure 1:**
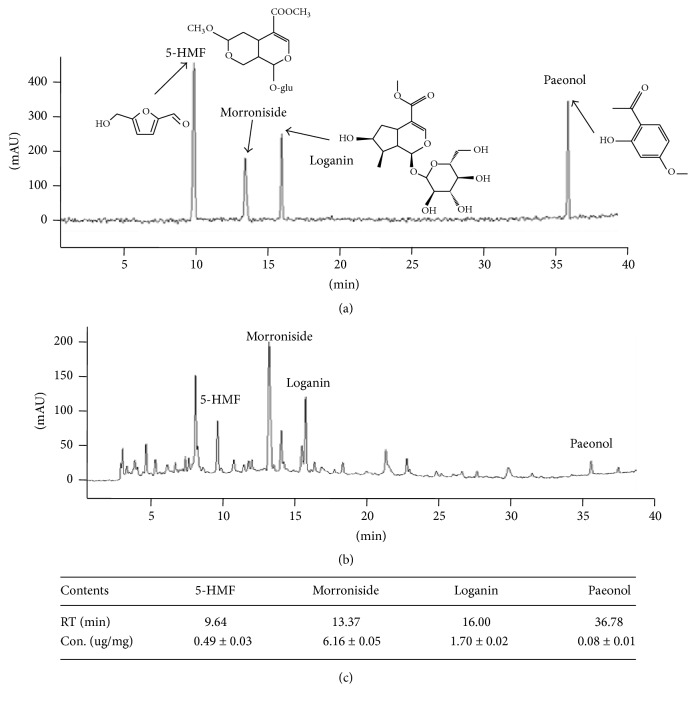
*Fingerprinting analysis of YJT and its reference compounds.* For fingerprinting analysis for YJT, YJT and its major reference compounds were subjected to the high-performance liquid chromatography (HPLC). Two-dimension histogram of YJT (a) and its four major compounds (b). Quantitative analysis of five reference components in YJT (c).

To verify the identification of used herbs and reproducibility of YJT preparation, the fingerprinting was performed using high-performance liquid chromatography-diode array detector-mass spectrometry (HPLC-DAD-MS) for YJT and its four reference compounds as follows:* Rehmannia glutinosa *Liboschitz* var*.* purpurae* Makino versus 5-hydroxymethyl-2-furfural (5-HMF),* Cornus officinalis* Sieb. et Zucc versus loganin and morroniside, and* Paeonia moutan *Sims versus paeonol, which were followed by the our previous conditions [21]. Briefly, after the dissolution (20 mg of YJT and 2 mg of each of the six herbal extracts in 1 mL of water; 0.01 mg of 8 standards in 1 mL of water or 50% methanol) and filtration, these formulations were subjected to HPLC analysis of Agilent 1100 series (Agilent Technologies, Santa Clara, CA). The HPLC system consisted of a SCL-10A system controller, LC-10AD pump, SPD-10MVP diode array detector, and CTO-10AS column temperature controller (Shimadzu, Kyoto, Japan). A Phenomenex Prodigy C18 (4.6 × 250 mm; particle size 5 *μ*m; Phenomenex, Torrance, CA) column was eluted with solvents A (10% acetonitrile in water containing 0.1% formic acid) and B (DW) at a flow rate of 0.4 mL/min. Solutions of 15% A and 85% B were changed to 60% B for 30 min, 40% B for 40 min, and 0% B for 60 min (Figures 1(a)–1(c)).

### 2.3. Animals and Experiment Plan

A total seventy of specific pathogen-free C57BL/6J male mice (12 weeks old, 24–26 g) were procured from Orient Bio (Gyeonggi-do, South Korea). The mice had free access to food pellets (Orient Bio, Gyeonggi-do, Korea) and water ad libitum and were kept in an animal room (temperature at 23 ± 2°C and humidity for 60 ± 5%, with a 12 h:12 h light and dark cycle). After 7 days of acclimatization, the mice were randomly divided into six groups (*n* = 10 for normal and *n* = 12 for other of each treatment groups): normal (no restraint stress with DW), Sco. Only group (scopolamine injection with DW), YJT groups (scopolamine injection with 50, 100, or 200 mg/kg), and Tac. group (scopolamine injection with tacrine 12.5 mg/kg). The YJT and tacrine were dissolved in DW and orally administered using dosing gavage for 7 consecutive days. On the 7th day of the experiment, all the mice were intraperitoneally injected to neutral saline (0.9%) or scopolamine (2 mg/kg), 1 h prior to Morris waster maze task at the first day. The Morris waster maze task was complete after further 4 consecutive days.

All the animal experiment procedures were approved by the Korea Food Research Institute (Gyeonggi-do, South Korea). The animal experiment was conducted in accordance with the Guide for the Care and Use of Laboratory Animals published by the United States National Institutes of Health (NIH).

### 2.4. Morris Water Maze Task

The Morris water maze task was performed according to the previous method [22]. Briefly, a circular pool (100 cm diameter 3 × 50 cm height) with a circular acrylic platform (10 cm diameter 3 × 35 cm height) was used and location of platform can be discriminated by visual cue. The pool was filled with milk water (22 ± 1°C) and divided into equal quadrants [23]. Data were recorded using a video camera connected to the corresponding software (Smart Junior, Panlab SL; Barcelona, Spain). Mice were placed on the platform for 10 s and removed from the pool. Mice were given acquisition trial for 4 days. The escape latency and cumulative path-length were recorded during each acquisition trial. On day 5th, all mice were subjected to probe trial without platform and were recorded for 120 s. The time spent in the target quadrant was measured for spatial learning and memory.

### 2.5. Preparation of Brain Tissue and Serum Samples

All mice were sacrificed under ether anesthesia condition after 1 h following the Morris water maze. Whole blood samples were isolated via abdominal vein and serum was collected by centrifugation at 3000 rpm for 15 min at 4°C. The hippocampal region was isolated from the whole brain tissue immediately, and then samples were stored at −80°C or in RNAlater (Ambion, TX). In each group, two mouse brains tissues were fixed in 4% paraformaldehyde (PFA) for immunohistochemistry (IHC) analysis after cardiac perfusion. Hippocampal areas in each hemisphere of remaining eight to ten mice were used for other experiment including biochemical analysis, western blot, and real-time PCR analysis. The part of hippocampus was homogenized on ice using radio-immunoprecipitation assay (RIPA) buffer and other parts of hippocampus were used for isolation of RNA.

### 2.6. Assessments of Laboratory Assays

For the determination of oxidative stress parameters and antioxidant component assays in the hippocampal area, the part of stored hippocampal tissue homogenates was used (*n* = 8 to 10 in each group). All assays were measured by commercial kit.

The lipid peroxidation was determined by the commercial product of Lipid Peroxidation (MDA) Colorimetric/Fluorometric Assay Kit (Catalog#, K739, Bio Vision, Milpitas, CA). The final products of MDA were measured at 530 nm using a spectrophotometer (Soft Max, Ver. 5.4, Molecular Devices, Sunnyvale, CA). Total glutathione (GSH) content was determined using a commercial kit (OxiSelect™, Catalog# STA-312, Cell Bio Labs, INC. San Diego, CA) with the absorbance measured at 405 nm using a spectrophotometer (Soft Max, Ver. 5.4, Molecular Devices). SOD activities were determined using an SOD assay kit (Dojindo Laboratories, Kumamoto, Japan), and dilutions of bovine erythrocyte SOD (St. Louis, MO) ranging in concentration from 0.01 to 50 U/ml were used as standards. Catalase activities were determined using a commercial kit (OxiSelect™, Catalog# STA-314, Cell Bio Labs, INC.). All procedures were carried out, according to the manufacturer's protocol. The absorbance was measured at 450 nm using a spectrophotometer (Soft Max, Ver. 5.4, Molecular Devices).

### 2.7. Western Blot Analysis

Each hippocampal area from whole brain tissues was homogenized in RIPA buffer with proteinase inhibitor solution for performance of western bloat analysis (*n* = 8 to 10 in each group). Protein samples (concentration was about 30 *μ*g to each sample) were subjected to sodium dodecyl sulfate polyacrylamide gel electrophoresis (SDS-PAGE) and transferred to polyvinylidene fluoride (PVDF) membranes (Millipore, Billerica, MA). The membranes were then incubated in PBS containing 5% nonfat powdered milk and 0.1% Tween 20 for 1 h to block using nonspecific binding before being incubated with primary antibodies for 4-HNE (#ab46545, Abcam, Cambridge, MA), iNOS (sc-7271, Santa Cruz Biotechnology Dallas, TX), and *β*-actin (sc-1616, Santa Cruz Biotechnology, Dallas, TX) overnight at 4°C in blocking solution (5% skimmed milk in 0.1% Tween 20 in 10 mM PBS, pH 7.3). The blots were washed and incubated with HRP-conjugated secondary antibody for 1 h at room temperature, and the peroxidase activity was detected using the Immobilon Western HRP detection reagent (Millipore) using an Image Reader (Thermo Fisher Scientific, Rockford, IL). The *β*-actin was used as a reference protein for all the results. The ratio of the protein of interest was subjected to *β*-actin.

### 2.8. Gene Expression Analysis

For analysis of the expressions of eight genes, the total RNA was isolated from hippocampal area (*n* = 4 to 5 in each group) using an RNeasy Mini Kit (QIAGEN, Valencia, CA). The cDNA was synthesized by using a cDNA synthesis kit (Invitrogen, Waltham, MA). The real-time PCR was performed using SYBR Green PCR Master Mix (Roche), and the PCR amplification was performed using a standard protocol with the IQ5 PCR Thermal Cycler (Bio-Rad, Hercules, CA). The detected genes for the gene expression analysis include the following: brain-derived GSH syntheses (GSS), GSH-reductase (Grd), GSH-peroxidase (Gpx), SOD-1, SOD-2, and SOD-3, muscarinic Ach receptor 1 (mAchR1), cAMP response element-binding protein (CREB), CREB-binding protein (CBP), and brain-derived neurotrophic factor (BDNF) were measured by real-time PCR. The primer sequences, product sizes, and annealing temperatures are summarized in Table 2. For analyzing the mRNA expression results using calculated the fold changes, the GAPDH was used as a reference gene.

### 2.9. Determination of AChE Activities and Brain-Derived Neurotrophic Factor (BDNF) Contents in Hippocampal Area

Acetylcholinesterase (AChE) activities and BDNF contents were measured in hippocampus using commercial colorimetric AChE activity assay kit (#ab138871, Abcam, CA, and #ab212166, Abcam) according to the manufacturer's protocol.

### 2.10. Determinations of IHC Analysis in the Brain Tissue

In other measurements of neuronal cell proliferation in the hippocampal area, the Ki67 staining was performed, and for determining lipid peroxidation, a final product of oxidative stress, the 4-HNE staining was also performed. At the final day of experiment, two mice in each group were progressed by cardiac perfusion using 4% PFA solution until blood was appeared clearly (200 to 300 mL of solution). After perfusion process, brain tissues were isolated and cryoprotected in the state of gradient sucrose solutions (10, 20, and 30%) for 24 h, embedded in tissue-freezing medium with liquid nitrogen, and cut into coronal frozen sections (40 *μ*m) using a cryostat.

Cryosections of each brain tissue were moved in to microscopy slide and were subjected to endogenous peroxidase quenched with 3% H_2_O_2_ in PBS (pH 7.3). And then tissues were treated by blocking buffer (5% normal chicken and goat serum in PBS for overnight at room temperature, RT), incubated with primary antibodies using 4-HNE (1 : 125, #ab48506, Abcam), and then washed using PBS (pH 7.3). After discarding the primary antibody, tissues were further incubated with a biotinylated goat anti-rabbit secondary antibody (1 : 250, #ab64256, Abcam). The tissues were exposed to an avidin-biotin peroxidase complex (Vectastain ABC kit, Vector) for 2 h. The peroxidase activity was visualized using a stable diaminobenzidine solution. Immunoreactions were observed using a microscope circumstance under the magnification of ×200 (Olympus, Germany) and quantified using the Image J 1.46 software (NIH, Bethesda, MD).

### 2.11. Statistical Analysis

All obtained data were expressed as mean ± standard deviation (SD). Statistically significant differences between the groups were analyzed by one-way analysis of variance (ANOVA) followed by post hoc multiple comparisons with Bonferroni *t*-test using the IBM SPSS statistics 20.0 (SPSS Inc., Chicago, IL). Differences at *p* < 0.05 were considered statistically significant.

## 3. Results

### 3.1. Fingerprinting Analysis of YJT

To obtain the chemical composition of the YJT, we subjected it to the HPLC equipment following the same conditions of our previous study [21]. We finally detected a total 4 of the reference compounds from YJT. Firstly, we identified 5-HMF which is a reference compound of* Rehmannia glutinosa *Liboschitz* var*.* purpurae* Makino at 9.46 min of retention time and its concentration was 0.49 ± 0.03 *μ*g/mg. Two of the compounds, such as loganin and morroniside, were detected from* Cornus officinalis* Sieb. et Zucc. at the retention time of 13.37 and 16.00 min, and the concentration was 6.16 ± 0.05 *μ*g/mg and 1.70 ± 0.02 *μ*g/mg, respectively. The paeonol was detected from* Paeonia moutan *Sims. in 36.78 min, and its concentration was 0.08 ± 0.01 *μ*g/mg (Figures 1(a)–1(c)).

### 3.2. Effects of YJT on the Memorial Dysfunction Analysis Using Morris Water Maze Task

**Figure 2:**
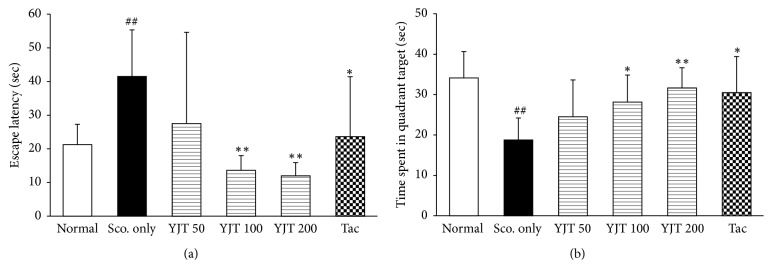
*Effects of YJT on the spatial learning and memory functional analysis using the Morris water maze.* The escape latency (a) and time spent in the target quadrant (b). Data are expressed as the mean ± SD (*n* = 10 to 12). *p*^##^ < 0.01 versus normal group; *p*^*∗*^ < 0.05 and *p*^*∗∗*^ < 0.01 versus scopolamine only injection group.

To estimate the pharmacological effects of YJT on the memorial dysfunction, we performed the Morris water maze test for 4 consecutive days for adaptable trials. At that day of 5th, scopolamine injection significantly caused to increase the escape latency, and the time spent in the target quadrant was significantly reduced by single injection of scopolamine as compared with normal group (*p* < 0.01 for both parameters, Figures 2(a) and 2(b)). On the other hand, preadministration with YJT (100 and 200 mg/kg) significantly improved both the escape latency (*p* < 0.01) and time spent in the target quadrant compared with the scopolamine only group (*p* < 0.05 or *p* < 0.01, Figures 2(a) and 2(b)). Preadministration with tacrine (12.5 mg/kg), used as positive drug in the current study, displayed similar effects of YJT.

### 3.3. Effects of YJT on the Hippocampal Protein Levels of GSH Contents, MDA, and Activities of Catalase and SOD

**Figure 3:**
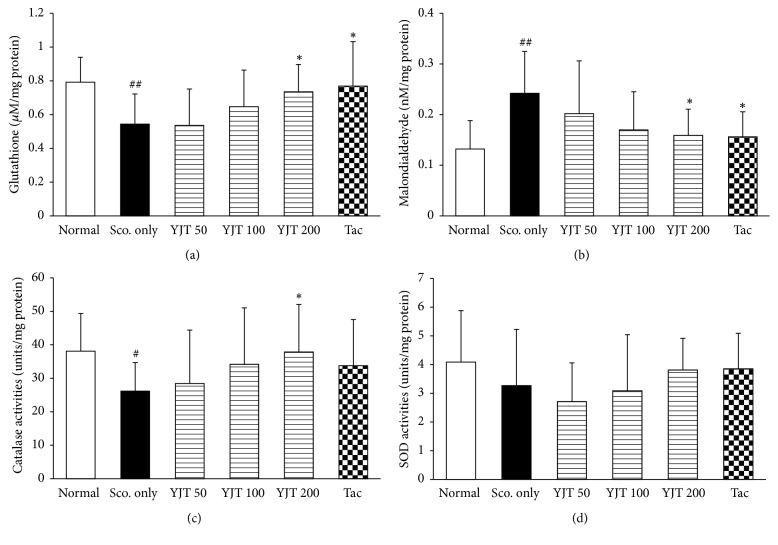
*Effects of YJT on the hippocampal protein levels of oxidative stress and antioxidant-related molecules.* Hippocampal tissue protein levels of glutathione (a), malondialdehyde (b), catalase activities (c), and SOD activities (d). Data are expressed as the mean ± SD (*n* = 8 to 10). *p*^#^ < 0.05 and *p*^##^ < 0.01 versus normal group; *p*^*∗*^ < 0.05 versus scopolamine only injection group. SOD: superoxide dismutase.

Regarding oxidative stress and antioxidant components, scopolamine aggravates the redox status imbalance in hippocampus especially. Thus, we determined the redox status by determinations of GSH contents, MDA, and activities of catalase and SOD, respectively. Hippocampal protein level of total GSH was decreased as 32%, but MDA was increased around 88% as compared with normal group. Those abnormal alterations by scopolamine injection were significantly normalized by preadministration with YJT compared to the scopolamine only group (*p* < 0.05 in 200 mg/kg, Figures 3(a) and 3(b)). These effects were well coincided with catalase activities in hippocampal area. The catalase activities were considerably decreased as 68% of normal group, whereas by preadministration with YJT (*p* < 0.05 for 200 mg/kg, Figure 3(c)), the SOD activities were not influenced by both scopolamine injection and YJT (Figure 3(d)).

Tacrine also considerably exerted to reduce the oxidative stress and ameliorate antioxidant components, but not SOD activities (Figures 3(a)–3(d)).

### 3.4. Effects of YJT on the Hippocampal Regions of 4-HNE and iNOS in Protein Levels and Gene Expression Levels of Antioxidant Components

**Figure 4:**
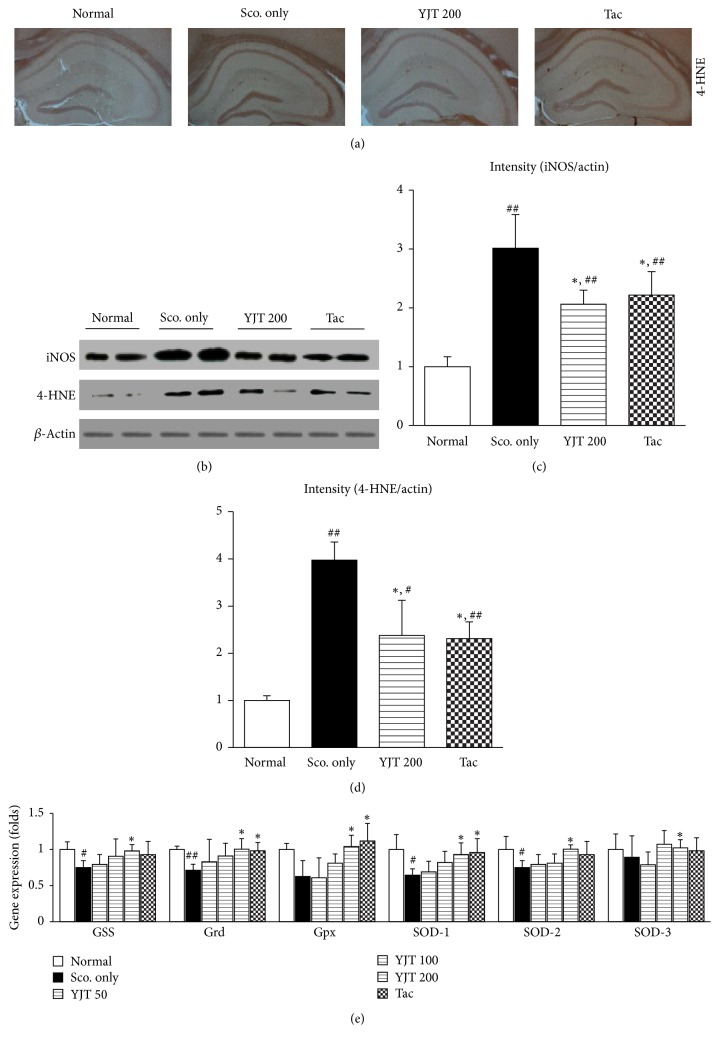
*Immunohistochemistry, western blot analysis, and gene expression analysis of redox status in the hippocampal area.* Immunohistochemistry analysis against the 4-HNE (a). Western blot analysis of iNOS and 4-HNE in the hippocampal regions (b) and their protein intensities (c, d). Gene expression analysis of antioxidant-related molecules including GSS, Grd, Gpx, SOD-1, SOD-2, and SOD-3 (e). Data are expressed as the mean ± SD (*n* = 2 for ICH analysis, *n* = 8 to 10 for western blot analysis, and *n* = 4 to 5 for gene expression analysis). *p*^#^ < 0.05 and *p*^##^ < 0.01 versus normal group; *p*^*∗*^ < 0.05 versus scopolamine only injection group. 4-HNE: 4-hydroxyneal, GSS: glutathione-synthase, Grd: glutathione-reductase, Gpx: glutathione-peroxidase, and SOD: superoxide dismutase.

To verify the antioxidant effects of YJT against scopolamine-induced hippocampal injury in the current model, we further examined IHC against to the 4-HNE antibodies. As we expected the positive signals (shown as deep brown color) in hippocampal areas were notably enhanced by scopolamine injection, and preadministration with YJT (particularly 200 mg/kg) significantly ameliorated those abnormalities (Figure 4(a)). Western blot analysis also well supported the pharmacological properties of antioxidant. The iNOS and 4-HNE in the hippocampal protein levels were markedly increased after being faced with scopolamine injection approximately 3.0- and 4.0- fold and theses oxidative injuries compared to normal group. Preadministration with YJT, however, significantly reduced those abnormal augmented of oxidative stress injuries as normal levels (*p* < 0.05 for 200 mg/kg, Figures 4(b)–4(d)).

Next, we performed the real-time PCR to investigate the degrees of antioxidant abilities in the gene expression level in the hippocampal regions. The GSS, Grd, Gpx, SOD-1, SOD-2, and SOD-3 were significantly lowered than that of normal group approximately 0.75-, 0.62-, 0.71-, 0.65-, 0.75, and 0.9-fold, respectively, as compared with normal group. However, pretreatment with YJT significantly normalized the above alterations as a normal level (*p* < 0.05, Figure 4(e)).

The similarities of YJT were also occurred by preadministration with tacrine in IHC against to 4-HNE signals and western blot analysis (*p* < 0.05, Figures 4(a)–4(d)), and tacrine also led to normalization of Grd, Gpx, and SOD-1 gene expression levels (*p* < 0.05, Figure 4(e)).

### 3.5. Effects of YJT on AChE Activities and BDNF Contents

**Figure 5:**
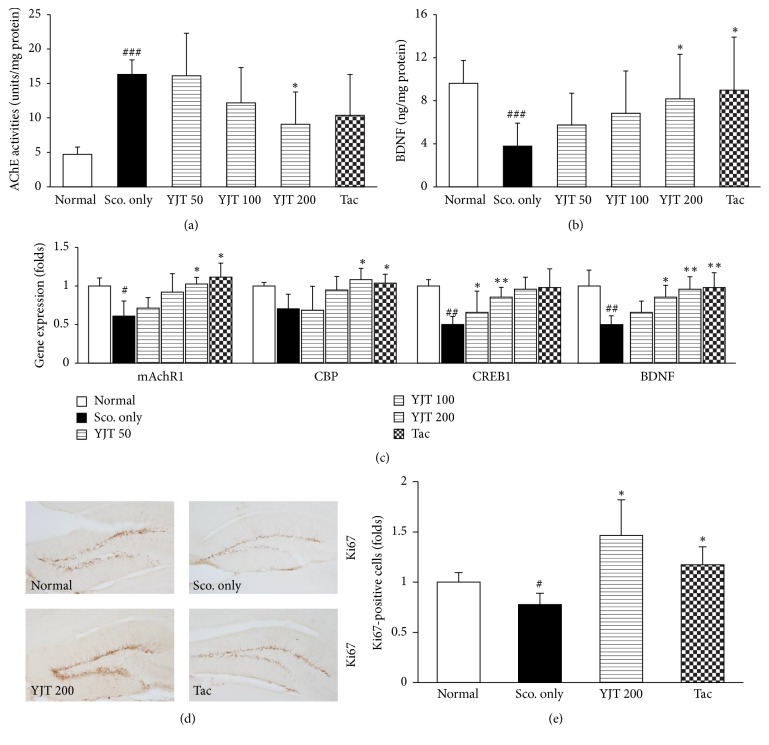
*Biomolecular analysis of memorial dysfunction and learning enhancement.* AChE activities analysis (a), BDNF contents (b), gene expression analysis of mAchR1, CBP, CREB1, and BDNF (c). Analysis of IHC against Ki67 (d) and its immunoreaction density (e). Data are expressed as the mean ± SD (*n* = 8 to 10 for AChE activities analysis and BDNF concentration; *n* = 4 to 5 for gene expression analysis; *n* = 2 for ICH analysis, resp.). *p*^#^ < 0.05, *p*^##^ < 0.01, and *p*^###^ < 0.001 versus normal group and *p*^*∗*^ < 0.05 and *p*^*∗∗*^ < 0.01 versus scopolamine only injection group. AChE: acetylcholinesterase, mAchR1: muscarinic acetylcholine receptor 1, BDNF: brain-derived neurotrophic factor, CREBP: cAMP response element-binding protein, and CBP: CREBP binding protein.

To investigate the possible mechanisms of YJT against the scopolamine injection-induced amnesia, we measured the hippocampal protein levels of AChE activity and BDNF. In the present study, we confirmed that the scopolamine considerably caused approximately 3.5-fold increases of AChE activities compared to the normal group. Preadministration with YJT (200 mg/kg), however, significantly ameliorated the abnormal elevations of AChE activities in hippocampal regions (*p* < 0.05, Figure 5(a)).

The BDNF, which is known for improving both the learning abilities and neurogenesis, in the hippocampal region contents was markedly lowered as 0.4-fold compared to that of normal group, whereas preadministration with YJT significantly recovered as normal levels compared to the scopolamine only injection group (*p* < 0.05, Figure 5(b)).

Tacrine group had similar effects of YJT in the present study (Figures 5(a) and 5(b)).

### 3.6. Effects of YJT on Neuronal Cell Proliferation Related Molecules

To verify the pharmacological mechanism of YJT properties on the memory function enhancement, we further examined the gene expression analysis of memorial function related molecules including mAchR1, CBP, CREB1, and BDNF and estimated the neuronal cell proliferation for IHC against Ki67 antibody.

The gene expression levels of mAchR1, CBP, CREB1, and BDNF were considerably downregulated in scopolamine only injection group as 0.61-, 0.71-, 0.5-, and 0.5-fold compared to normal group, whereas these abnormal alterations were significantly normalized as normal level by preadministration with YJT as normal levels (*p* < 0.05 or *p* < 0.01 in YJT 100 or 200 mg/kg, Figure 5(c)).

Regarding the neuronal cell proliferation, the positive cells of the positive cells of Ki67 staining were significantly decreased approximately 0.7-fold due to scopolamine injection group as compared with the normal group, whereas preadministration with YJT (200 mg/kg) significantly recovered those of Ki67 positive cells in dentate gyrus as compared with the scopolamine only injection group (*p* < 0.01, Figures 5(d) and 5(e)).

The preadministration with tacrine also showed similar effects on the gene expression levels of mAchR1, CBP, and BDNF, and Ki67, respectively (*p* < 0.05 or *p* < 0.01, Figures 5(c)–5(e)).

## 4. Discussion

In the current study, we answered our questions and proved them based on the scientific experiments whether the YJT efficiently protected a single scopolamine injection-induced memory deficits using C57/BL 6 mice model. Furthermore, we proved the pharmacological properties of YJT regarding on the Ach system modulated signaling pathways mainly.

First of all, we observed the therapeutic effects of YJT on the memory deficit, which is induced by scopolamine injection, using Morris water maze test. As our expectation, YJT significantly ameliorated the memory deficit as evidence by improvement of both the time spent of quadrant target and escape latency (Figures 2(a) and 2(b)). As a key organ of memorial reorganization and its functions, next we further explored to prove the possible pharmacological mechanisms of YJT.

The major possible pathophysiological mechanisms of neurodegeneration diseases are deeply linked to the central cholinergic system alterations, including cholinergic neurons, neurotransmitters, and their receptors [9, 14]. Thus, the therapeutics access is mainly focused on the inhibition of AChE and N-methyl-D-aspartate (NMDA) receptors for treating Alzheimer's disease currently [24, 25]. For proving them, we next measured the AChE activities in the hippocampal area in the brain tissue. As our expectation, the scopolamine injection considerably elevated AChE activities, whereas YJT efficiently block it (Figure 5(a)). Besides, the AChE activity is also closely linked to the cyclic adenosine monophosphate/protein kinase A- (cAMP/PKA-) CREB signaling pathway via G-coupled protein receptors [26]. Under the condition of excessive AChE activities by itself, this also can lead to provoking ACh disruption especially in hippocampal cholinergic synapses [27]. Moreover, the gene expression levels of mAChR-138 were considerably depleted to the patients with Alzheimer's disease that is well coincided with memory disruption in a mAChR-1 knockout mouse model [28, 29]. In the current study, we partially proved the YJT effects above them in the hippocampal area by performance on the gene expression analysis (Figure 5(c)). In our study, YJT also efficiently improved the deterioration of BDNF in protein and gene expression levels of hippocampal tissues (Figures 5(b) and 5(c)).

On the other hand, CREB, which is well known to the transcription factor of BDNF, is essential molecule for working of memory and synaptic plasticity in the CNS. If phosphorylated-CREB in the hippocampal region is disrupted, it can augment neurodegenerative diseases [30]. Furthermore, previous studies well showed that the CREB play roles as neuroprotective properties on the oxidative stress-induced neuronal damage model [31]. Thus, CREB activation is most critical issue to ameliorate cognitive impairment on the therapeutic access of neurodegenerative diseases [32]. In the current study, we partially proved the YJT effects on the above them in the hippocampal area by performance on the gene expression analysis (Figure 5(c)). In our study, YJT also efficiently improved the deterioration of BDNF in protein and gene expression levels of hippocampal tissues (Figures 5(b) and 5(c)). Regarding the enhancement of learning ability and neurogenesis, BDNF directly leads to improve them via phospho-CREB signaling pathways. The IHC staining against the Ki67 well supported the above results (Figure 5(d)).

Additionally, brain tissue is well known to susceptibility to the oxidative damage, and previous reports well evidenced the relevance between oxidative stress and neurodegenerative diseases. Thus, we further examined the antioxidant effects of YJT scopolamine injection-induced neurodegenerative model in the current study. The total GSH content in the hippocampal area was significantly recovered by YJT treatments, and it is also notably exerted to decrease MDA levels, which is a final product of lipid peroxidation (Figures 3(a) and 3(b)). The GSH is a predominant antioxidant peptide that directly quenches to oxidize-protein or peptide adducts especially oxidative stress damage condition. This potent antioxidant plays a pivotal role for maintaining homeostasis between oxidative stress and antioxidant. Regarding the enzymatic-antioxidant we observed that the catalase activities were significantly increased by YJT treatments (Figure 3(c)), but not SOD. Interestingly, both the scopolamine and YJT treatments did not affect the SOD levels in the hippocampal areas (Figure 4(d)). Evidenced by numerous of previous studies, brain tissue generally consumes high rate of oxygen, has relatively low amount of antioxidant components [33], and contains abundant amount of polyunsaturated fatty acids [34], transition metal irons, and high sensitivity of blood brain endothelial cells [35]. The above previous studies are well comprised of the antioxidant properties of YJT.

In the TKM theory, which has been practiced for thousand years, the main reasons of the excess stress are evoked from the imbalance between* “Qi*” and* “blood”* streams, the two of essential components of the human body. Based on the above theory, the YJT has been popularly prescribed for treating patients with neurodegenerative diseases such as memory deficit, mild senile dementia, anxiety, or depression over than hundreds of years in clinical practice [36]. Besides, previous studies also are well reflected regarding antiamnesic effects of the YJT. The potent components of herbal plants from the YJT such as Alpiniae oxyphyllae Fructus* (Alpinia oxyphylla Miq)* and Schizandrae Fructus* (Schisandra chinensis Baillon)* showed pharmacological effects on the brain damage using animal based* in vivo* models [37–39].

Although we firstly proved the pharmacological properties of YJT on the scopolamine-induced dementia mice model, the limitation remained. In the current study, we used the scopolamine to induce neurodegenerative disease-evoked memory deficit condition, but this drug can only partially mimic some extent of memory impairments such as mild-to-moderate Alzheimer's disease due to cholinergic system disruption [40]. It can not entirely represent the senile plaque and tau accumulations, which are hallmarks of Alzheimer's dementia. Therefore, we need to investigate the neuroprotective effects of YJT using another potent animal model such as in near future [41]. Next, we still unclearly know what kind of component in YJT could be positively worked to the antineurodegenerative effects. Thus, further study will be needed to figure out which individual herbal medicine or its derived chemical components will be effective or not based on the fingerprinting analysis data.

## 5. Conclusion

In the current study, we investigated the antioxidant properties of the YJT under conditions of single injection of scopolamine-induced brain memory deficit of C57/BL6 mice model which can mimic the pathological condition of neurodegenerative diseases. The possible mechanisms of the YJT were improvement of antioxidant components as well as enhancement of Ach activity signaling pathways. The further, however, study will be needed to examine the effects of YJT in the clinical trial.

## Figures and Tables

**Table 1:** Components of *Yuk-Mi-Jihwang-Tang *and its ratio.

Herbal name	Specific name	Used amount (g)
Rehmanniae Radix Preparata	*Rehmannia glutinosa *Liboschitz* var. purpurea *Makino	200
Dioscoreae Rhizoma	*Dioscorea japonica *Thunb.	100
Corni Fructus	*Cornus officinalis *Sieb. et Zucc.	100
Moutan Cortex Radicis	*Paeonia moutan *Sims.	75
Alpiniae oxyphyllae Fructus	*Alpinia oxyphylla *Miq.	75
Schizandrae Fructus	*Schisandra chinensis *Baill.	50

Total amount	-	600

**Table 2:** Sequence of the primers used in real-time PCR analysis.

Gene list (NCBI number)	Primer sequencing (forward and reverse)	Product size (base pair)	Annealing temperature (°C)
GSS (NM-001291111)	5′-ACC GAA GGC TGT TTA TGG ATG A-3′	100	59
	5′-AGG CGT GCT TCC CAG TTC T-3′		
Grd (NM-010344.4)	5′- GAT GTG TGG AGC GGT AAA CTT TT -3′	120	60
	5′- AGC CGC CRG AAC ACC ATC TA -3′		
Gpx (NM-001329527.1)	5′-CTC ACC ATT CAC TTC GCA CTT C-3′	122	59
	5′-ACA CCA GGA GAA TGG CAA GAA-3′		
SOD-1 (NM_011434)	5′-TGT CAG GAC AAA TTA CAG GAT TAA CTG-3′	100	60
	5′-AAA TGA GGT CCT GCA CTG GTA CA-3′		
SOD-2 (NM_013671)	5′-CCCAGACCTGCCTTACGACTAT-3′	112	58
	5′-GGTGGCGTTGAGATTGTTCA-3′		
SOD-3 (NM_011435)	5′-GGT GGA TGC TGC CGA GAT-3′	101	59
	5′-GCT GCC GGA AGA GAA CCA A-3′		
SOD-3 (NM-011435)	5′-GGT GGA TGC TGC CGA GAT-3′	101	59
	5′-GCT GCC GGA AGA GAA CCA A-3′		
mAChR 1 (NM-001112697)	5′-AGT GGC ATT CAT CGG GAT CA-3′	100	60
	5′-CTT GAG CTC TGT GTT GAC CTT GA-3′		
CBP (NM-001025432)	5′-CTG GCA GAC CTC GGA AAG AA-3′	100	59
	5′-CTG GCG CCG CAA AAA CT-3′		
CREB 1 (NM-013497)	5′-ACA GTG CCA ACC CCC ATT TA-3′	100	59
	5′-GTA CCC CAT CCG TAC CAT TGT T-3′		
BDNF (NM-001048139)	5′-CAC TTT TGA GCA CGT CAT CGA A-3′	104	60
	5′-CAC CCG GGA AGT GTA CAA GTC-3′		
GAPDH NM_001289726	5′-TCA CTC AAG ATT GTC AGC AAT GC-3′	100	58
	5′-GGC CCC GGC CTT CTC-3′		

BDNF: brain-derived neurotrophic factor, CREBP: cAMP response element-binding protein, CBP: CREBP binding protein, GSS: glutathione synthase, Grd: glutathione-reductase, Gpx: glutathione-peroxidase, mAchR1: muscarinic acetylcholine receptor 1, SOD: superoxide dismutase.
